# Neurodiversity: post-cognitivist foundations of the 3E approach for educational inclusion of autistic students with technology

**DOI:** 10.3389/fnhum.2024.1493863

**Published:** 2025-02-03

**Authors:** Ronnie Videla, May Britt Aros, Francisco Parada, Leonie Kausel, Eduardo Sandoval-Obando, Daniela Jorquera, David Ibacache, Sebastián Maluenda, Pablo Rodríguez-Herrero, Carola Cerpa, María Jesús González, Marcelo Chávez, Paola Ramírez

**Affiliations:** ^1^Escuela de Educación Diferencial, Universidad Santo Tomás, La Serena, Chile; ^2^Innova STEAM Lab, La Serena, Chile; ^3^Escuela de Educación, Universidad Católica del Norte, Coquimbo, Chile; ^4^Centro de Estudios en Neurociencia Humana y Neuropsicología, Facultad de Psicología, Universidad Diego Portales, Santiago, Chile; ^5^Escuela de Psicología, Instituto Iberoamericano de Desarrollo Sostenible, Universidad Autónoma de Chile, Temuco, Chile; ^6^Escuela de Psicología, Universidad de La Serena, La Serena, Chile; ^7^Escuela de Formación del Profesorado, Universidad Autónoma de Madrid, Madrid, Spain; ^8^Facultad de Ciencias Básicas, Universidad Católica del Maule, Talca, Chile

**Keywords:** neurodiversity, autism, post-cognitivism, 3E approach, inclusive education, technology educative, smart devices, virtual reality

## Abstract

The concept of neurodiversity has gained strength in the last years to highlight the value of individual differences based on relevant variations in brain functioning. Inclusive education has embraced neurodiversity to promote a culture centered on valuing diversity, in response to clinical models based on deficits or disorders. This theoretical-critical article argues for the need to complement the current foundations of neurodiversity with post-cognitivist perspectives that reaffirm the brain-body-environment continuum, in order to enrich inclusive educational practices for autistic individuals. We begin by reviewing and discussing the concept of neurodiversity and neurocentric arguments in light of post-cognitivism. We then explore the potential of the 3E Cognition approach (embodied, enacted, and environmentally scaffolded) for addressing autism, aiming to provide a holistic understanding that contributes to the practical application of cognitive neuroscience findings in inclusive education. Finally, we present some guidelines and practical cases for creating inclusive educational environments based on digital technologies that enhance agency and sensory multimodality for autistic students.

## Introduction

Many curricula worldwide adhere to educational inclusion projects to ensure the learning of diverse children, using the concept of neurodiversity to foster the acceptance and inclusion of all people while also valuing neurological differences (General Teaching Council for Scotland, 2020; Milton et al., 2020). Neurodiversity seeks to raise awareness of the potential of diversity and difference, countering clinical perspectives based on ideas of disorders and deficits, such as Autism Spectrum Disorder (ASD), Attention Deficit Hyperactivity Disorder (ADHD), Dyslexia, and Learning Disabilities (LD) (Nilsson et al., 2019). Neurodiversity aims to reframe educational inclusion and the richness of diversity from an ethical and rights-based perspective, particularly for individuals considered socially or cognitively atypical, such as those with autism (Pripas-Kapit, 2020). Within the framework of Sustainable Development Goal 4, which references quality education based on equity and justice, schools aim to create inclusive environments where autistic individuals can learn curriculum content in various ways, minimizing the misalignments and dysregulations they face with insipid information-processing methodologies (OECD, 2018; UNESCO, 2022; Varsik and Vosberg, 2024).

The hegemonic transmission model of conventional schools, based on memorizing discourses and concepts detached from context and the body, has constrained and distorted the role of agency and sensory multimodality in educational design with materials and technologies (Videla and Veloz, 2023). Much of the evidence on inclusive education for autistic individuals has primarily focused on addressing the dysregulation of sensory information processing, language, and social interaction that restrict adaptive capacity in prototypical environments (Brede et al., 2017). Furthermore, the hyper- and hypo-behavioral reactions of autistic children in classrooms are often challenging for teachers to address due to the limited university training in psychoeducational strategies and the use of technologies for neurodiverse individuals (Fletcher-Watson and Happé, 2019). The challenges of inclusive education for autistic individuals include a broader understanding of neurodiversity, based on contemporary cognitive foundations that adhere to a relational ontology emphasizing the role of the body and context (Grohmann, 2017; Delafield-Butt, 2021). This is aimed at enriching inclusive educational practices by creating environments that resonate with the capabilities of autistic individuals, using diversified teaching strategies, as well as incorporating contemporary technologies: Artificial Intelligence, Robotics, Augmented Reality and Virtual Reality (Valencia et al., 2019).

In this article, we discuss two fundamental aspects that contribute to the theoretical foundations of a holistic understanding of the education of autistic individuals and the practical application of neuroscience for this same purpose. The first aspect involves incorporating post-cognitivist approaches, such as 3E Cognition (embodied, enacted, and environmentally scaffolded), which highlight the brain-mind-environment interplay (Parada et al., 2024). We begin by emphasizing the reductionist critique derived from the prefix “neuro,” which often reduces the complexity of experience to neuronal dynamics, functional anatomical structure, and neurotransmitters. Subsequently, given that our application is educational, and therefore a relational phenomenon embedded in dynamic cultural and sociomaterial environments, we argue for the potential of the 3E approach in revaluing (i) the body, (ii) agency with materials, and (iii) artifacts. The second aspect involves the use of contemporary technologies that support the inclusion of autistic individuals, with the goal of providing teachers with tools to scaffold content comprehension processes and behavior regulation beyond Universal Design for Learning (UDL). In doing so, we recognize the complexity of autism, aiming to contribute to the scientific discussions on neurodiversity that support inclusive education in making informed decisions on cognitive, didactic, and technological foundations (Toro et al., 2020).

### Rethinking neurodiversity within the framework of post-cognitivism

The notion of neurodiversity refers to the diversity of all individuals, considering these differences as relevant variations in brain function (Béné, 2023). Neurodiversity is generally associated with individuals with Autism Spectrum Disorder (ASD), Attention Deficit Hyperactivity Disorder (ADHD), Dyslexia, and Learning Disabilities, as well as other neurological or developmental variations linked to learning disabilities. It also extends to aspects of social and sexual diversity (Dwyer, 2022). The neurodiversity movement emerged with the goal of promoting the acceptance and inclusion of all people while simultaneously affirming differences. This approach responds to deficit-based models, where students are labeled based on what they cannot do rather than what they can contribute to. Currently, within the framework of inclusive education, the term Autism Spectrum Condition (ASC) is preferred over ASD. This initiative by the neurodiverse community, from an ethical and rights-based perspective, aims to replace the clinical and pathological view with a more inclusive and valuable understanding of difference (Lai et al., 2013; Ozonoff, 2012; Baron-Cohen, 2015).

While neurodiversity emphasizes the natural variability of human brain structures and functions as a strength for educational inclusion, the prefix “neuro” implies a reductionism of cognition, learning, and ultimately teaching, focusing primarily on the brain (Slaby, 2010). There is currently a lack of attention on the constitutive role of the brain-body-environment assembly in understanding neurodiversity. It is essential to counteract this aspect in order to advance the practical application of neuroscience in education, particularly concerning the design of diversified teaching with materials and technologies that foster environments suitable for autistic individuals. The case of Universal Design for Learning CAST (2018) is relevant for exemplifying neurocentrism as the foundation of inclusive educational practice, which also influences neurodiversity, by suggesting that teachers plan according to brain areas based on information processing. Specifically, we advocate for more holistic perspectives, such as SpEED (Special Education Embodied Design for Sensorially Equitable Inclusion), which incorporates the sensorimotor engagement of the body with the world as a fundamental axiom for inclusive design (Abrahamson, 2014; Tancredi et al., 2021).

Within the framework of post-cognitivism, the view of information processing as being confined to brain processes occurring in the head is challenged by contemporary evidence from cognitive neuroscience (Kelso, 1992; Fuchs, 2017; Noë, 2010), the 4E Cognition approach—”embodied, enacted, embedded, extended” (Newen et al., 2018; Parada and Rossi, 2020)—and updated ecological psychology (Gibson, 1979; Chemero, 2009; Heft, 2018), all of which advocate for the notion of cognitive systems. The idea of cognition as a process of symbol manipulation has generated a set of detractors who oppose internalist reductionism and, therefore, the mind-body (Penny, 2023) and thought-action dualism (Shapiro and Stolz, 2019). These notions are rooted in Maturana and Varela’s (1973) biology of knowledge concerning the dynamic continuity of mind and life through the concept of autopoiesis, which provides neurobiological foundations that challenge the apparent distinction between organism and environment (Thompson, 2007). These foundations rest on structural determinism and the history of sensorimotor couplings as the ontological basis of cognition, where each living organism possesses a unique structure that enables it to perform actions in accordance with its characteristics and dynamic relationship with the environment (Varela et al., 1991; Di Paolo et al., 2017).

The current perspective of 3E Cognition (embodied, enacted, and environmentally scaffolded) is a simplified version of the 4E approach, appealing to the practical dimension of those applying contemporary cognitive principles, steering clear of the radical philosophical tensions that often characterize cognitive scientists (Parada et al., 2024). The 3E perspective contributes to the application of neuroscience and cognition to the real world. Rather than seeking behavior in the relationship between brain structure and cognitive function, the post-cognitivist paradigm adheres to a more complex and holistic view of the integrated functioning of various systems in the organism (Gallagher, 2023). The 3E approach resonates with arguments from brain ecology that emphasize its role as a mediator and integrator, contributing to the regulation of organism-environment interactions through ascending, descending, and horizontal chains of neurophysiological activity. This leads to the formation of dynamic networks of neuronal assemblies and activity patterns that extend throughout the brain, distributing according to contexts of mechanistic integration among enabling and contextual factors across different systems (central nervous, vestibular, skeletal, circulatory, and muscular) (Parada and Rossi, 2018). These ideas align with the concept of the entangled brain, where networks do not function independently but are “intertwined” and operate in an integrated manner to support cognition, through overlapping functions, where the same networks can participate in different tasks as a result of contextual modulation (Pessoa, 2023).

### 3E cognition: toward a post-cognitive understanding of autism

ASD is a heterogeneous neurodevelopmental spectrum that is characterized by challenges in social communication and the presence of repetitive and restrictive interests. ASD can develop with or without intellectual disability, language impairment, sensory difficulties or other comorbidities (American Psychiatric Association (2013). Neuroscience studies on autism have found that there are structural and functional differences in the brains of individuals with ASD, which are understood to contribute to ASD symptomatology (Sato and Uono, 2019; Kausel et al., 2024; Soto-Icaza et al., 2024). But these findings are often hard to transfer to real-world settings that could benefit psychologists, occupational therapists, special education teachers, and educators in their daily practice with individuals with ASD. They may wonder: How can these findings from the specialized neuroscience community support inclusive education with materials and technologies for students with autism?

A post-cognitive argument to support broader studies in neurodiversity, which facilitate discussion for informed educational decision-making, involves those conducted in real-world contexts. Theoretical proposals that use technologies such as Mobile Brain/Body Imaging/4E (Grasso-Cladera et al., 2023) and use them in classroom settings (Parada, 2018) could help to reinforce that the organism’s body and all the (neuro)physiological states that emerge from being in a natural, sociomaterial, and culturally grounded technological world are integrally connected to the organism’s experience (Parada and Rossi, 2018). These ideas align with contemporary approaches like 3E Cognition, which highlight the role of environmental scaffolding in developing practices that resonate with students’ multimodal experiences, such as solving real-world problems using challenging technologies and educational approaches (Parada et al., 2024).

Critical reflection on the notion of neurodiversity within the framework of a holistic understanding of autistic individuals is grounded in contributions to inclusive educational design through the generation of diversified teaching strategies using various materials and technologies justified by environmental scaffolding. This involves revitalizing the role of agency and the sensorimotor dynamics that underlie cognition and learning. The 3E Cognition approach has catalyzed more precise initiatives to address, among other areas, the transfer of neuroscience to education (Parada et al., 2024). In the case of neurodiversity, which emphasizes an inclusive view that values brain differences, we advocate for a broader valuation that integrates. brain-body-environment. Otherwise, neurocentrism and a more systemic disconnection between the organism and environment prevail—for example, by reducing behavior to purely cerebral explanations of sensory processing (Dunn, 1997) that emphasize the role of thalamic connectivity and serotonin and GABA receptors (Ayub et al., 2021). While these neurological aspects are crucial for understanding enabling mechanisms, it is essential to complement this evidence with studies that provide guidance on situated sensory processing. Such guidance is pertinent for teachers to benefit autistic students through diversified educational experiences that minimize the brain-body-environment mismatch (Jones et al., 2020). These guidelines, which address enabling mechanisms or contextual factors, range from classroom lighting, multimodal perception educational materials, to the use of technologies that channel pedagogical challenges, preventing hyper- and hypo-sensory adverse reactions (Piller and Pfeiffer, 2016; Moon et al., 2024).

While this argument is informative and useful for understanding the brain’s role in learning processes and dysregulation in autistic individuals, it is restrictive for the inclusive educational community that guides practice, considering the complexity of the organism at molecular, sensory, and relational levels (Fuchs, 2017). If neurodiversity aims to value individual differences, these differences must be articulated within a holistic framework. To this end, the 3E approach plays a fundamental role in considering autism within an integrated brain-body-environment system, where the differences are not only within the brain but in the constitution of mechanisms that dynamically synchronize enabling organisms and enabling contextual factors (Rossi et al., 2019). Whether the context is school, family, psychotherapy, or the natural environment, it is more pertinent to assume that autism can be a variation of a diverse sensory experience in a body and environment, rather than merely a diversity of the brain. While experience arises with the brain, it does not cause it. This is known as the mereological fallacy (Craver and Bechtel, 2007; Gallagher, 2018).

The 3E approach supports these claims, assuming that environmental scaffolding occurs through the co-creation of participatory meaning, such that recurrent interactions and the use of materials and technologies can maximize or diminish the learning potential of autistic individuals. This is because learning does not depend solely on brain variation but on the constitutive dynamics of the brain-body-environment system. Likewise, this approach aligns with the studies of Cadena Alvear and Gastelum Vargas (2022), who have applied post-cognitive principles from enactivism to critique the notion of autism as a deficit in the ability to “mentalize” other mental states, often linked to Theory of Mind, as well as being the product of a socio-emotional and executive deficit, see also De Jaegher (2013). Additionally, Szokolszky and Kékes Szabó (2019) have reaffirmed this broader conception of cognition in individuals with autism, emphasizing the role of sensorimotor processes and perception-action, as well as arguments that social relationships are interdependent processes during development that dynamically influence each other.

Given that cognition is a relational phenomenon, the 3E approach does not require questioning whether the cognitive agent’s ability is causally linked to the environmental, implemented, or structured scaffold. Instead, the ability is a transient, multidimensional constitutive factor of the sensorimotor coupling between brain, body, and environment (Parada et al., 2024). For this reason, the behavior of autistic individuals should be understood beyond reductionist explanations of neuronal dynamics and functional anatomical variability. Rather, environmental characteristics should be considered as enabling factors that modulate cognition, depending on the types of interactions with the environment, such as in inclusive education (Bertolotti and Magnani, 2017; Charalambous and Djebbara, 2023).

### Use of technologies in the educational inclusion of autistic individuals

The 3E approach provides fertile ground for designing diversified strategies that also incorporate technology for autistic individuals. In this context, learning begins with the embodied resources that students already possess. These include prior sensorimotor experiences, practices, processes, and skills. Thus, teaching should be flexibly adapted to the sensorimotor diversities of the students, since different bodily engagements with the world shape cognitive structures (Fincher-Kiefer, 2019). The sensorimotor differences among students can influence how they interact with what they are learning, making the use of technologies and materials essential for providing diversified teaching strategies (Hasan and Nene, 2024).

The generalized educational approach to addressing neurodiversity in educational systems, such as UDL (Universal Design for Learning), is based on three design principles for learning derived from cognitive neuroscience: recognition networks (multiple means of representation), strategic networks (multiple means of action and expression), and affective networks (multiple means of engagement) (Rose and Meyer, 2002). Recognition networks help categorize information (located in posterior lobes of the neocortex), strategic networks (located in the frontal lobes) are responsible for thoughts and ideas, and affective networks (located in central neocortex and limbic system) foster enthusiasm and motivation (Rose and Lapinski, 2011). These neurocentric ideas that guide inclusive educational practice can be seen in claims about the potential of UDL with learning technologies: “indeed, there are as many facets or types of learning as there are divisions in the brain” (Dolan et al., 2013:14). In contrast, we emphasize that environmental scaffolding and sensorimotor perception are just as important as brain structures. In the case of autism, contemporary technologies offer a diverse range of support for teachers in their inclusive educational efforts through environmental scaffolding. These technologies include digital devices, robots, and sophisticated wearables designed to address sensory regulation, social interactions, and stereotyped behaviors (Grynszpan et al., 2014; Lane and Radesky, 2019).

**FIGURE 1:**
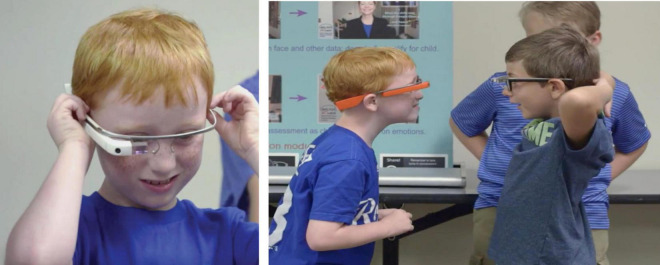
The Brain Power System is the world’s first Augmented Reality Smart-Glass-System to empower children and adults with autism to teach themselves crucial social and cognitive skills. In these images, authorized by Dr. Ned Sahin, children can be seen using Brain Power’s Smart-Glass System in open social interaction contexts.

The use of technologies has proven to be a valuable tool in enhancing interaction among autistic children, considering the varying levels of social motivation, social development, and interest in digital technologies (Laurie et al., 2021). This, because that inclusive design should aim to create systems that are accessible and adaptable to a wide range of users, including those with diverse neurological profiles (Tamura et al., 2019). Brain Power (see Figure 1) is a digital technological system created by Dr. Ned Sahin (MIT neuroscientist) that runs on Smartglasses, aimed at improving the social and cognitive skills of autistic children and adults. This technology leverages augmented reality (AR) and artificial intelligence (AI) to deliver neuroscience-based, gamified digital learning. It was developed as a wearable device using Google Glass, along with a suite of applications designed to assist with daily activities, and a web-based dashboard for tracking and measuring progress through Affive, an emotion-detecting AI. The feasibility and effectiveness of this system was reported in several articles published in Sahin et al. (2018) from MIT, including a single-case study of a child with ASD (Keshav et al., 2018), and pilot studies of children with ASD (Vahabzadeh et al., 2018a) and children and young adults with ASD (Vahabzadeh et al., 2018b). The interventions ranged from a single session up to 3 week sessions, and were well rated by educators/caregivers, indicating that the system was easy to implement and use, and that the experience was fun for the students. Also, importantly, in general results showed a decrease of irritability, hyperactivity, and social withdrawal symptoms in the participants as reported by educators/caregivers after the interventions.

From a 3E approach, the smart glasses can be considered environmental scaffolds, allowing children and adolescents to enhance their multimodal perception of coupling with the social environment by adjusting to social cues, including the ability to focus on facial and eye regions when desirable. Since this application measures physiological signs of stress and attention direction through AI, it can provide augmented reality cues and images that help recalibrate perceived mismatches in various activities. Figure 1 shows children using the smart glasses in open contexts, highlighting their non-invasive use. Additionally, as wearable digital devices embedded in visual perception, they offer greater bodily dynamism compared to tablets or phones, which require looking down. The glasses encourage social interaction by keeping hands free and allowing users to remain engaged with their sociomaterial environment.

**FIGURE 2:**
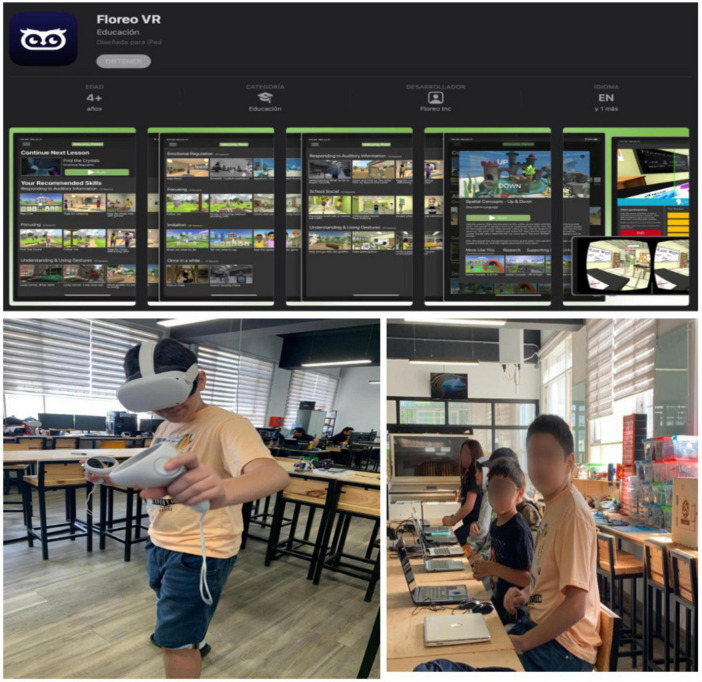
Floreo is a learning app that leverages virtual reality to teach science-based social and communication skills to individuals with Autism Spectrum Disorder. This image shows a Level 2 autistic student from our summer STEAM education academy engaging with Floreo’s virtual reality application, demonstrating a preference for virtual reality over other technologies in the lab.

Other immersive technologies, such as virtual reality (VR) and video games, offer a practical and effective approach to diversifying inclusive pedagogical strategies with digital technologies for autistic individuals, with a primary focus on social communication, including social functioning, emotion recognition, and speech and language (Dechsling et al., 2021; Zhang et al., 2022). VR-based training provides an enriched immersive environment for autistic individuals, using its applications to scaffold cognitive and emotional skills (Coninx and Stephan, 2021). A relevant case in this field involved the creation of 3D design solutions in immersive environments and block-based programming, inspired by the observation of bridges in natural settings (Moon et al., 2024). Autistic students participated in various phases of the virtual reality-based training, where they were tasked with designing a 3D drawbridge within a virtual environment that simulated a realistic island ecosystem (see Figure 2). Based on a situated problem involving the urgent need to transport food onto the island for survival, the students took on the role of engineers, tasked with finding the best design solution for the bridge. They had to consider factors such as height, structural integrity, and how well the bridge would integrate into the transport network. The students, represented by avatars, used virtual reality tools to build the bridge piece by piece (foundations and pillars) and then simulated the bridge’s functionality using block-based programming. This process facilitated hands-on engagement with different forms of environmental scaffolding enabled by task diversification in the virtual space, all focused on the same goal: “bridge construction.”

The study’s results showed statistically significant differences in task performance, solution effectiveness, and the incorporation of social feedback, with those participating in the immersive environment outperforming those who did not. From a 3E approach, autistic students tend to prefer and excel in immersive environmental scaffolds, which help them connect their design ideas, programming skills, and social learning relationships. Similarly, this type of environment can be framed within an inclusive digital ecosystem that provides diverse ways to teach (3D design, programming, and virtual reality) and learn how to think with different digital technologies (Aguayo et al., 2023). The specific experience, as well as the problem-solving challenges, allows autistic students to embody diverse forms of learning that become more sophisticated through practice with tools embedded in challenging immersive environments. As part of our STEAM (Science, Technology, Engineering, Art and Math) education summer schools of 2024, we encountered a 12-year-old child with level 2 autism who demonstrated intellectual development comparable to that of an 8-year-old. The child exhibited repetitive behaviors, limited social interaction, and socioemotional dysregulation during collaborative challenges involving project-based learning workshops that integrated 3D modeling and fabrication technologies, AI, Arduinos, and virtual reality. The child’s primary interest was centered on the VR glasses that were available in the laboratory, which were used in various contexts beginning with coordination games and progressing to the use of the Floreo app.

Virtual reality applications like Floreo (Ravindran et al., 2019) offer a range of educational environments with activities that teachers and families can implement at school or at home. Unlike shared real-world settings, where students with special educational needs must continuously interact and follow a thematic continuity, virtual reality designed through relational engineering (Gapenne et al., 2024) provides alternative possibilities that support, facilitate, regulate and enhance learning in diverse environments (Videla et al., 2024). As such, there is a fertile ground for research to find the best ways of contributing to the learning process of people with ASD and ID using these technologies. Floreo is a virtual reality platform specifically designed for autistic individuals to practice diverse simulated skills such as self-regulation, attention, and social interaction, developed collaboratively by medical professionals, therapists, engineers, and neurodiverse individuals (National Institute of Mental Health, 2025). This VR environment provides a safe space for learners to acquire social, behavioral, communication, and life skills that can be applied in daily life. It also addresses the needs of individuals with ASD and Attention Deficit Hyperactivity Disorder (ADHD) and other neurodiverse conditions, demonstrating its potential to support personalized and inclusive educational experiences.

The use of virtual and augmented reality technologies in educational contexts presents specific challenges when applied to autistic individuals, due to the highly individualized nature of their sensory processing and personal experiences with these tools. Personalized evaluations are essential, focusing on elements such as colors, sounds, the presentation of digital content, and the sense of presence in immersive contexts, as these factors may trigger sensory dysregulation or aversive reactions. Occupational therapy specialists profile autistic students based on sensory processing, social interaction, and emotional regulation characteristics, enabling personalized educational designs that avoid reinforcing learning barriers through homogeneous approaches. Augmented reality, for instance, offers opportunities to enhance perception through 3D digital overlays in natural environments, supported by user co-design and interdisciplinary collaboration, aligning with students’ specific interests. Similarly, virtual reality requires bespoke, gradual designs that encourage exploration and adaptation while reducing sensory overload. Given the social demands of traditional classroom settings, virtual reality environments can provide self-directed, interest-driven learning experiences that are more inclusive and responsive to the individual needs of autistic students.

## Conclusion

Neurodiversity is a promising approach that promotes educational inclusion by recognizing the value of different brains. However, we believe that these differences should be framed within a holistic understanding that integrates the brain, body, and environment (Gallagher, 2023). The primary reason lies in the practical implications cognitive neuroscience can bring to systemic fields like inclusive education, whose goal is to provide diverse learning opportunities for autistic individuals. While learning is inevitable, the core of education is that students always learn for particular motivations, from someone, and usually through something (Biesta, 2024).

Inclusive education often faces challenges within a classroom ecology that includes sensory-diverse individuals, such as autistic students, who require greater pedagogical effort to scaffold various contents through discourse, materials, and technologies. From a post-cognitivist perspective, we reaffirm the continuity of brain, body, and environment, as it is more relevant for teachers to understand the cognition of autistic individuals through their active engagement with their surroundings, rather than making forced and biased inferences from technical neuroscientific evidence into the educational field. To this end, we adopt the 3E cognition approach (embodied, enacted, and environmental scaffolding), which provides an ontological and epistemological framework that highlights the role of environmental scaffolding in the variety of interactions between the agent and the environment, as it facilitates, supports, or regulates cognition and learning (Varga, 2019).

From a 3E perspective, we see significant limitations in the idea that educational technology should be guided by specific brain areas. This approach tends to fragment, generalize, and minimize the role of inclusive education with technology, as seen in the case of UDL, which is based on brain networks that guide design principles focused on knowledge, expression, and engagement. If diversity is only viewed in terms of brain function, then the means and context for learning become irrelevant. Agency, along with the many ways individuals can interact with technology and materials, is diminished by a prescriptive curriculum and linear pedagogy that focuses on specific learning pathways for sensory-diverse individuals. This is especially concerning when evidence highlights the importance of diverse materials and dynamic digital ecosystems for autistic individuals (Cardy et al., 2023). In this perspective article, we advocate for environmental scaffolding from a multimodal cognition viewpoint, where sensory diversity expands through practical engagements and enriched educational environments with people, materials, and technology (Malafouris, 2013).

We agree with Tancredi et al. (2022) that the design of inclusive educational technology should incorporate post-cognitivist approaches that foster diversified practices, useful for creating new contexts that emphasize the body and the environment. Technologies such as “Brain Power” smartglasses, which integrate augmented reality, artificial intelligence, and emotional stress monitoring, can positively impact the quality of social relationships and emotional self-regulation in autistic individuals by serving as environmental scaffolds that guide visual perception based on personal experiences (Colombetti and Krueger, 2015). Similarly, virtual reality-based technologies can create diversified teaching strategies for autistic students in virtual environments. In these settings, students can utilize digital tools like 3D design and block-based programming to cultivate cognitive and affective skills linked to problem-solving in situated activities. From this perspective, the technologies employed can be considered environmental scaffolds that help regulate and redirect cognitive activity through the embodiment and embedding of thought in immersive environments. This is highly valuable for inclusive education using technology, as both smartglasses and virtual reality-based training offer significant support to teachers who face daily challenges in interacting with autistic individuals.

Brain Power’s Google Glass and Floreo VR offer promising tools for educational scaffolding in various contexts, such as schools and homes, particularly for children and adults with ASD. Google Glass can support activities like emotion recognition, where the Emotion Charades app helps children identify and respond to facial expressions through visual or auditory feedback modeled by a facilitator. It can also aid in practicing visual attention during group tasks, using features like Face2Face to encourage eye contact and collaboration, and foster collaborative learning through guided cues for turn-taking and rule-following during games. Similarly, Floreo VR provides immersive, engaging, and affordable lessons designed to enhance emotional regulation, focused attention, and social interaction. These experiences are recommended for children aged 7–11 under supervision by teachers or family members to monitor behavior and ensure effectiveness. However, caution is necessary as Floreo VR is not suitable for children under 7 years old or individuals with conditions such as a history of seizures, photosensitivity, ocular movement disorders like strabismus, migraines, or susceptibility to motion sickness. Virtual reality use may cause symptoms like dizziness, vertigo, headaches, nausea, sweating, or fatigue, and its use should be discontinued if such discomforts arise. These considerations emphasize the need for tailored, supervised implementation of immersive technologies to ensure safety and maximize educational benefits.

As future guidelines for an inclusive educational curriculum with contemporary technologies, it is recommended to incorporate various forms of environmental scaffolding that include challenging activities focused on tacit knowledge and emotional self-regulation, similar to those implemented in Australia’s interoceptive curriculum (Goodall and McAuley, 2019). The incorporation of new technologies, along with their design principles and the implementation of diversified resources and strategies, not only requires the adoption of digital solutions but also a renewal of cognitive paradigms (Nathan, 2021). This shift strengthens the ecological foundations of inclusive educational practice with technology within the framework of neurodiversity, in light of contemporary 3E Cognition approaches.

## Data Availability

The raw data supporting the conclusions of this article will be made available by the authors, without undue reservation.
